# Probing Long Non-coding RNA-Protein Interactions

**DOI:** 10.3389/fmolb.2017.00045

**Published:** 2017-07-11

**Authors:** Jasmine Barra, Eleonora Leucci

**Affiliations:** ^1^Laboratory for Molecular Cancer Biology, Department of Oncology, KU Leuven Leuven, Belgium; ^2^Center for Cancer Biology, VIB Leuven, Belgium

**Keywords:** RNA, RBPs, pull-down assays, RNA immunoprecipitation, crosslinking

## Abstract

Non-coding RNA sequences outnumber the protein-coding genes in the human genome, however our knowledge of their functions is still limited. RNA-binding proteins follow the transcripts, including non-coding RNAs, throughout their life, regulating not only maturation, nuclear export, stability and eventually translation, but also RNA functions. Therefore, development of sophisticated methods to study RNA-protein interactions are key to the systematic characterization of lncRNAs. Although mostly applicable to RNA-protein interactions in general, many approaches, especially the computational ones, need adjustment to be adapted to the length and complexity of lncRNA transcripts. Here we critically review all the wet lab and computational methods to study lncRNA-protein interactions and their potential to clarify the dark side of the genome.

## Introduction

Over the past decade extensive efforts have been made to refine our understanding of the most complex mystery of life: the genome. With the recent advent of deep sequencing methodologies, it has become clear that the full range of the protein-coding and non-protein-coding transcriptome is still vastly underestimated (Rinn and Chang, 2012). We now know that the eukaryotic genome is pervasively transcribed, but the protein-coding genome (that includes around 20,000 genes), only accounts for 2% of all sequences (Carninci et al., 2005). The so called “dark matter” of the genome gives rise to a broad spectrum of processed and regulated transcripts that do not appear to code for functional proteins, but seem to participate in a variety of biological processes (Fatica and Bozzoni, 2014; Huarte, 2015; Lorenzen and Thum, 2016).

The largest group of ncRNAs arbitrary includes all the transcripts that are over 200 nucleotides, namely long non-coding RNAs (lncRNAs). The class is very heterogeneous and despite the progresses made in annotating them, lncRNA diversity makes it impossible to draw sweeping generalizations or predict their molecular mechanisms. LncRNAs can localize to nuclear (e.g., XIST; Engreitz et al., 2013) or cytoplasmic fractions (e.g., chl1; Carrieri et al., 2012) or even shuffle between the two compartments (Montes et al., 2015) where they can exert very different functions. Moreover, being transcribed by RNA Pol II, they are often 5′-capped, spliced and polyadenylated, and thus very similar in structure to mRNA (Quinn and Chang, 2016).

However, compared to mRNAs, lncRNAs are less efficiently spliced (Schlackow et al., 2017) and generally display modest sequence conservation (Chodroff et al., 2010; Necsulea et al., 2014) which led to dismiss them as transcriptional noise (Quinn and Chang, 2016). Nevertheless, the lack of conservation can easily be explained by the fact that lncRNAs are free from codon preservation constraints, whereas secondary and tertiary structures may play an important role (Ponjavic et al., 2007; Chodroff et al., 2010).

Another point raising doubts about the functionality of lncRNAs is the lack of clean genetic models, showing evidence for phenotypic changes upon perturbation of their expression. So far only a handful of genetic *in vivo* models exist, showing developmental defects (Sauvageau et al., 2013; Standaert et al., 2014), or effects on tumorigenesis (Yildirim et al., 2013; Adriaens et al., 2016; Arun et al., 2016). This is partly due to the tedious procedures needed to obtain appropriate knock-out mice, and partly to the lack of conservation of many lncRNAs. Hopefully these questions will now be efficiently tackled thanks to new technologies such as the CRISPR/Cas9 for genome manipulations.

Another crucial step toward the demonstration of lncRNA functionality is the dissection of the molecular pathways in which they are involved. To this end, a successful approach is the characterization of their interactors, especially (but not only) their protein partners.

During and after transcription, RNAs are subjected to multiple processing and regulatory steps that are coordinated by dynamic interactions with other nucleic acids and by a variety of RNA-binding proteins (RBPs) (Moore, 2005). RBPs often follow RNAs throughout their life, regulating maturation, nuclear export, stability and eventually translation (Gehring et al., 2017), therefore identifying RBP interacting with non-coding RNAs is important to understand their function.

**Figure 1:**
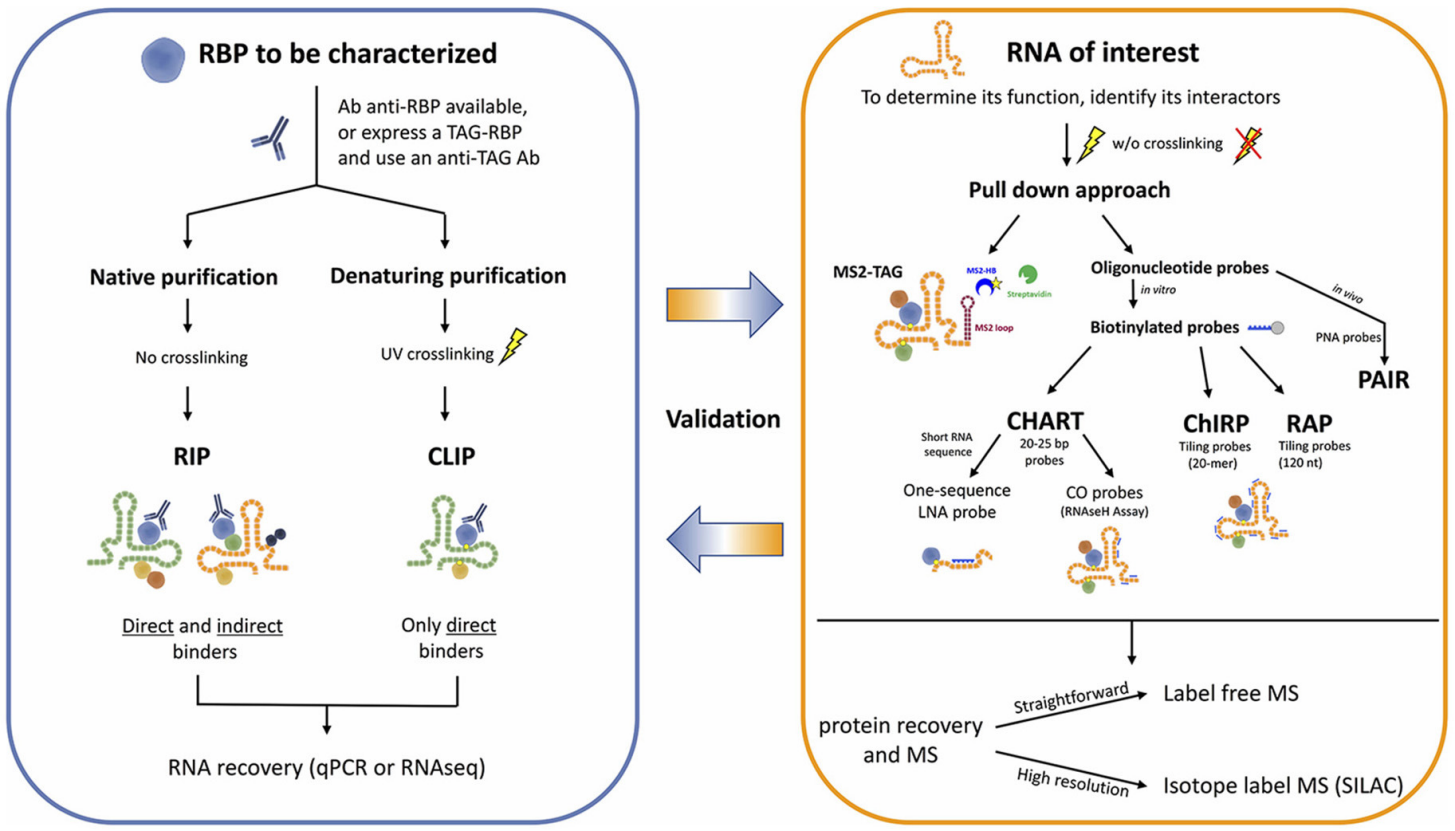
Workflow for the identification of RNA-protein interactions.

Development of new sophisticated methods to study RNA-protein interactions are enabling the systematic characterization of lncRNAs. These approaches can be generally classified in either RNA-centric or protein-centric (Figure 1). While the first ones aim at purifying an RNA of interest to identify the interacting proteins by mass spectrometry, the second ones rely on the purification of a specific protein to find the interacting RNA molecules, identified by RNA sequencing. Here we will discuss both approaches and the principal methods applied in the most recent exciting discoveries, highlighting their advantages and limitations (Tables 1, 2).

**Table 1:** Protein-centric approaches.

**Methods**	**PROS**	**CONS**
RIP	Performed under physiological conditions to preserve the native complexes Requires little specialized equipment and/or reagents	Relies on the availability of good antibodies, or the use of tagged RBPs Lacks high-stringency washes and crosslinking of RBPs to RNAs, which leads to low signal to noise ratio and frequent misinterpretations in the data analysis Additional control conditions may be required to distinguish true interactions from non-specific ones Does not determine the exact location of the binding site of RBPs
CLIP	Application of strong washing steps allows to get rid of non-specific binders	UV radiation can alter the RNP infrastructure, and crosslinking is not homogeneously efficient Low efficiency of UVC (254 nm) RNA-protein crosslinking Difficult identification of the exact site of crosslink within the sequenced fragment
HITS-CLIP	Genome-wide tool	The eluted RNA must be de-crosslinked, cDNAs are truncated at the crosslink site and get lost during the standard library preparation protocol
PAR-CLIP	Single nucleotide resolution to identify the exact site of binding of the RBP on the RNA (the nucleotide analogs are converted into cytosine (C) for 4-SU, or adenine (A) for 6-SG, and can be used to specifically mark the exact binding site)	The eluted RNA must be de-crosslinked, cDNAs are truncated at the crosslink site and are lost during the standard library preparation protocol Nucleotide analogs can be toxic for cells and animal models More expensive than the classic CLIP approach
iCLIP	Single nucleotide resolution to identify the exact site of binding of the RBP on the RNA	Needs special adaptors to allow the circularization step, not always highly efficient Input material required: high It relies on UV-C crosslinked peptide-RNA interactions to halt reverse transcriptase (RT); cDNA molecules generated in iCLIP strategies are, on average, shorter than the isolated RNA crosslinked to the RBP of interest
eCLIP	Can determine whether two identical sequenced reads come from two unique RNA fragments or from PCR duplicates of the same RNA fragment	Addition of adapters in 2 separate steps: it can be challenging and is a further source of variability during library preparation Input material required: high
irCLIP	Input material required: 100 lower than other CLIP approaches Simplified protocol (magnetic-bead-based purification and ‘two-tube’ strategy)	

**Table 2:** RNA-centric approaches.

**Methods**	**PROS**	**CONS**
ChIRP	Do not require a priori knowledge of the lncRNA domains involved in the interaction Cheap 20-mer probes that biophysically provide optimal discrimination against off targets	Oligonucleotides can potentially also directly pull down DNA fragments with sequence similarities, in an RNA-independent manner. Appropriate controls are thus needed to eliminate such background signal
RAP	Do not require a priori knowledge of the lncRNA domains involved in the interaction Longer tiling probes (120 nt), so that all potential hybridization spots are fully used	120 nucleotides probes can be considerably costly to synthesize
CHART	A minimal set of capture oligonucleotides probes may result in a reduced background	The identification of the accessible sites is done via RNAse H assay, a time consuming and tedious procedure Using non-tiling probes can be less efficient in hybridizing the lncRNA under study and additional controls may be needed

## Protein-centric methods

To unravel the mechanisms by which RNA binding proteins (RBPs) affect RNA processing and ncRNA functions, many technologies such as RNA immunoprecipitation (RIP) and cross-linking and immunoprecipitation (CLIP) have been important allies to identify the RNA substrates of RBPs, systematically and comprehensively.

The so-called protein-centric methods employ antibodies to immunoprecipitate the endogenous protein of interest and the associated RNAs. Many variants of these methods have been developed, but their essential difference is the condition of immunoprecipitation, either native or crosslinked.

The native purification methods started with RNA immunoprecipitation (**RIP**), eventually followed by high-throughput sequencing (**RIP-seq**) (Zhao et al., 2010), or microarray (**RIP-chip**) (Keene et al., 2006) for the identification of the associated RNA fragments, enabling a transcriptome-wide view of protein–RNA interactions. RNA-protein complexes are purified under physiological conditions to preserve the native complexes as much as possible. The strength of the RNA-RBP association is calculated as the enrichment of the RNA isolated by target-specific immunoprecipitation, compared to a control immunoprecipitation. AGO2 RIP followed by qPCR for instance, was used to demonstrate miRNA-dependent decay of MALAT1 in the nucleus of mammalian cells (Leucci et al., 2013). A similar approach but followed by microarray, was used by Khalil and colleagues to show that about 20% of lincRNAs associates with PRC2 complex (Khalil et al., 2009). The main limitations of RIP approaches are (i) the possible loss/gain of targets during the extraction, including non-specific interactions that may form after cell lysis (McHugh et al., 2014), (ii) the identification and exclusion of false positive hits, and (iii) the determination of the exact location of the binding site of RBPs that will require subsequent motif analysis (Li et al., 2015). To overcome these drawbacks, crosslinking of the RNA-protein complexes in the living cells was introduced. The dominant method for crosslinking RNA-protein complexes is treatment of cells with short wavelength UV light to induce the formation of covalent crosslinks only at sites of direct contact between proteins and RNA, but not between interacting proteins, specifically reducing the eventual artifacts (König et al., 2012).

The cross-linking and immunoprecipitation (**CLIP**) method was initially combined with the Sanger Sequencing method (Ule et al., 2003) and later with high-throughput sequencing (RNA-seq) (Chi et al., 2009; Darnell, 2012). Briefly intact cells are UV irradiated, to freeze RNA-protein interactions, RNA is partially digested (typically with RNAse A) and bound complexes are isolated via immunoprecipitation of a targeted protein along with the crosslinked RNA fragment. A key step allowed by the crosslinking is the application of strong washing steps to remove non-specific binders. Proteins are then digested and the RNA is converted to cDNA for sequencing. The main limitations of CLIP are the low efficiency of UVC (254 nm) RNA-protein crosslinking, and the difficult identification of the exact site of crosslink within the sequenced fragment. Therefore, distinction of true target RNA segments from background non-crosslinked RNA fragments can be quite challenging.

CLIP evolved quickly with the advent and development of **next generation high-throughput sequencing (genome-wide CLIP) technology** enabling the investigation of genome-wide RBP–RNA binding at single base-pair resolution. Genome-wide CLIP (CLIP-seq) (Wang et al., 2009) bears some similarities to RIP-seq, except that RIP-seq lacks high-stringency washes and crosslinking of RBPs to RNAs, which leads to low signal to noise ratio and frequent misinterpretations in the data analysis (Riley and Steitz, 2013). Recent digestion optimized RNA immunoprecipitation with deep sequencing (**DO-RIP-seq**) combined aspects of both RIP and CLIP to quantify the binding strength of different RNA binding sites, during dynamic biological processes. It can measure enrichment scores for RNA-protein binding, both at the whole target transcript level (RIP-Seq-Like or RSL) and at the binding site level (BSL) using continuous metrics, overcoming the above mentioned limitations of simple RIP approach (Nicholson et al., 2016).

We highlight below three major technologies for genome-wide CLIP experiments hereby briefly described and compared.

1. High-throughput sequencing of RNA isolated by UV-crosslinking and immunoprecipitation (**HITS-CLIP**) was developed as a genome-wide tool to map protein-RNA binding sites *in vivo*, by RNA-seq. For the library preparation a 3′ RNA adaptor needs to be ligated to the RNA fragments in order to allow reverse transcription using probes complementary to the 3′ adaptors (Chi et al., 2009; Licatalosi et al., 2009; Gillen et al., 2016). Using this technique, Cáceres lab showed that miRNAs are not the most abundant targets of DGCR8, which is instead mainly bound by snoRNAs and lncRNAs (Macias et al., 2012).

A variant of HITS-CLIP is Photoactivatable-Ribonucleoside-Enhanced Crosslinking and Immunoprecipitation (**PAR-CLIP**). The main difference is that cells are cultured in the presence of nucleotide analogs such as 4-thiouridine (4-SU) or 6-thioguanosine (6-SG) in the media, which is incorporated into the newly synthesized RNAs. UVA light (365 nm) exposure will crosslink the modified residues of the RNAs and the interacting proteins. During the reverse transcription step the nucleotide analogs are converted into cytosine (C) for 4-SU, or adenine (A) for 6-SG, and can be used to specifically mark the exact binding site at single nucleotide resolution (Riley and Steitz, 2013; Garzia et al., 2016; Yoon and Gorospe, 2016; Benhalevy et al., 2017). HITS-CLIP and PAR-CLIP are the most commonly used techniques for protein-RNA interaction studies, but they both suffer one main disadvantage. The eluted RNA must be de-crosslinked before cDNA library preparation and sequencing, and this is generally achieved by digestion with proteinase K. Unfortunately, this leaves a polypeptide fragment on the RNA, at the site of protein-RNA interaction, which causes premature truncation of the reverse transcription reaction. Hence, most cDNAs are truncated at the crosslink site and get lost during the standard library preparation protocol. A good example of PAR-CLIP applied to the lncRNA world is a paper by Kaneko and colleagues, where the authors describe a 30-amino-acid region in JARID2 responsible for the binding of many lncRNAs from the imprinted Dlk1-Dio3 locus, including MEG3. Binding of lncRNAs to JARID2 would then be necessary for the recruitment of PRC2 to the chromatin in embryonic stem cells (Kaneko et al., 2014).

2. The individual-nucleotide resolution CLIP (**iCLIP**) approach managed to overcome the loss of truncated fragments during library preparation by employing a self-circularization strategy and barcoding, thus strongly improving the quality of the quantitative information obtained from the sequencing (König et al., 2011). Using modified primers for the RT reaction, the truncated cDNA molecules can be marked with a cleavable adaptor and a barcode, allowing their self-circularization. The adaptor region is then cleaved at a specific site, and the linearized cDNA molecules can be entirely amplified. Each sequence will contain a barcode followed by the nucleotide that was crosslinked, and the exact position of the RNA-protein interaction can be determined with individual-nucleotide resolution (König et al., 2010). The main drawback of iCLIP is the efficiency in mapping small RT-PCR products. Optimal fragments range from 40–100 nt, and will generate uniquely mappable cDNAs inserts of 20–50 nt. Variation on the theme are eCLIP and irCLIP that try to optimize different aspects of the classic iCLIP approach. The enhanced CLIP (**eCLIP**) improves the library preparation step and optimizes the efficiency of the circular ligation step of the iCLIP by adding adapters in two separate steps. (i) An indexed 3′ RNA adapter is ligated to the crosslinked RNA fragment on beads during the immunoprecipitation, (ii) a 3′ single-stranded DNA adapter is ligated after reverse transcription, to determine whether two identical sequenced reads come from two unique RNA fragments or from PCR duplicates of the same RNA fragment (Van Nostrand et al., 2016b). Another more recent variation on the theme is the UV-C crosslinking and immunoprecipitation platform infrared-CLIP (**irCLIP**). By use of infrared-dye-conjugated and biotinylated ligation adaptors, this platform allows a rapid and at the same time quantitative analysis of *in vivo* captured protein–RNA interactions, using even less material than required by the other standard CLIP protocols (Zarnegar et al., 2016).

## Choosing the appropriate method and control conditions

A critical step in any experimental design is finding the balance between the goals of the study and the protocol requirements, therefore choosing the appropriate CLIP method is not trivial. Although the use of PAR-CLIP has been successfully reported not only on human cell lines, but also on entire organisms, such as *C. elegans* (Jungkamp et al., 2011) and *S. cerevisiae* (Freeberg et al., 2013), and even cell lines from other organisms like *D. melanogaster* (Xiong et al., 2015) or mice, it is very expensive and not always a viable option. Nucleotide analogs can in fact be toxic in alive animals and in this case HITS-CLIP would be a preferable setting. Nevertheless, for a higher resolution in determining binding sites, PAR-CLIP or iCLIP, although technically challenging, remain the ultimate approach (Wang et al., 2015).

All CLIP procedures are elaborate, multi-step procedures that require extensive optimization and proper controls. Bias can arise from several sources and we will hereby mention the major ones. (i) UV radiation allows harsh washings but carries also disadvantages: it can physically and chemically alter the ribonucleoprotein network, leading to results that are far from being physiological. UV cross-linking efficiency is a major issue as it shows sequence bias at the level of both RNA and protein. Generally greatest photoreactivity is observed between pyrimidines and amino acids such as Cys, Tyr, Trp, Phe, and Lys (Gaillard and Aguilera, 2008). (ii) The creation of RNA libraries is an extremely critical step as the efficiency is affected by the nucleotide composition of the RNA linkers, the PCR and the sequencing step itself (Hafner et al., 2010). (iii) Background controls are essential. A non-irradiated sample control for example represents the contaminants captured in a UV crosslinking–independent manner (Castello et al., 2015). The presence of a common background in PAR-CLIP datasets has been noted by multiple studies (Friedersdorf and Keene, 2014) and although computational tools exist to analyze CLIP-seq data, only few address the background issue (Comoglio et al., 2015; Reyes-Herrera et al., 2015; Wang et al., 2015). When possible, “positive controls” can be also useful, as in the PAR-CLIP approach the incorporation of photoactivatable ribonucleotides can be used as an internal control for crosslinking efficiency (Ascano et al., 2012). (iv) Extensive experimental replication will reduce systematic bias and increase the reproducibility of genome-wide CLIP experiments. (v) Also the extensive validation process is necessary to establish functionally relevant RNA-protein interactions (Jungkamp et al., 2011).

## RNA-centric methods

To determine the direct functions of lncRNAs the key pieces of information one may need are *where* they act, and with *whom* they interact. To address these questions, emerging technologies have been developed to generate genomic binding profiles of lncRNAs on chromatin, and/or to systematically study their protein interactome.

Almost concomitantly three ingenious approaches emerged in the field.

1. Capture Hybridization Analysis of RNA Targets (**CHART**) was initially developed as a hybridization-based purification strategy to map the genomic binding sites of endogenous RNAs and it was first used to determine the genome-wide localization of roX2, a 600-nt ncRNA that regulates dosage compensation in *Drosophila* (Simon et al., 2011). CHART is now commonly used also to purify lncRNA-associated proteins.
2. Pioneering work by the Chang lab brought to the development of a new method termed Chromatin Isolation by RNA Purification (**ChIRP**) that can be combined to deep sequencing and allow unbiased high-throughput discovery of RNA-bound DNA and proteins (Chu et al., 2012, 2015). The key turnaround was the use of short oligonucleotide probes tiling the length of an RNA of interest, a concept inspired by tiling arrays which provide specific signal and reduced off targets. A further expansion of ChIRP is a recent related technique that interrogates functional subunits of lncRNAs. Domain ChIRP (**dChIRP**) can iteratively find the minimal set of probes targeting the RNA of interest, map at the domain-level RNA–RNA, RNA-protein, and RNA-chromatin interactions as well as identifying genomic binding sites with high sensitivity. Biotinylated antisense 20-mer oligonucleotides are designed to hybridize with non-overlapping and non-redundant sequences on the RNA of choice. Cells are chemically crosslinked as in the standard CHART protocol, chromatin is sheared in small fragments and divided into equal samples to which oligonucleotide pools, all targeting different regions of the RNA of interest, are hybridized under stringent conditions. RNA, DNA and proteins can be separately recovered and analyzed, providing simultaneously information on RNA-, DNA-, protein-interacting domains of an RNA (Quinn et al., 2014).
3. RNA Affinity Purification (**RAP**) was initially developed by the Guttman lab to investigate the mechanisms of the Xist lncRNA localization during X-chromosome inactivation (XCI), a paradigm of lncRNA-mediated chromatin regulation (Engreitz et al., 2013). RAP is a sensitive approach to systematically map RNA-DNA interactions, and it can be adapted to specifically identify direct intermolecular RNA-RNA interactions (RAP-RNA) (Engreitz et al., 2014) or to explore RNA-protein interactions. A recent study successfully combined RAP with SILAC mass spectrometry to address the protein interactome of Xist during X Chromosome inactivation (XCI) in mouse ES cells (McHugh et al., 2015).

These RNA-centric approaches share common features as well as differences, mainly concerning the crosslinking method (reversible chemical-crosslink or UV crosslink) and the probe design, but depending on the RNA of interest, they can be all adapted, mixed and matched. For example *ad hoc* combination of RAP-MS and ChIRP-like-MS methodologies allowed efficient purification and characterization of *SAMMSON* and its interacting partners, revealing peculiar features of this melanoma specific lncRNA (Leucci et al., 2016).

The **probe design** is generally a critical step because the accessible areas of the target RNA might be unknown or hidden by secondary structures. Probes for CHART are empirically determined after RNase H assay to map the accessible regions on the RNA of interest. These regions will be targeted by specifically designed probes (Davis and West, 2015). On the other hand, both ChIRP and RAP do not require a priori knowledge of the lncRNA domains involved in the interaction since they tile oligonucleotides across the entire target RNA sequence trying to cover it as much as possible. Whereas ChIRP uses short 20-mer probes (Chu and Chang, 2016), RAP favors longer probes that are around 120-nucleotides (Engreitz et al., 2015). To avoid off targets the use of probe sets targeting multiple regions of the target RNA is generally preferred. Nevertheless, an alternative has been proposed where a single 25-bp sequence probe (targeting the RNA of interest SPRY4-IT1) was modified with a locked nucleic acid (LNA) backbone and a 5′-biotin label (Mazar et al., 2014).

Another type of modified probes are peptide nucleic acid (PNA) analogs used in the so called **“PAIR approach.”** PNAs and LNAs probes are more resistant to nuclease digestion and due to their higher melting temperature their interaction with the RNA target is highly specific, which makes them optimal to study specific mRNA spliced variants. The RBP–PNA complexes can be isolated by magnetic beads coupled to an antisense PNA oligo, and recovered proteins are then identified by mass spectrometry (Zielinski et al., 2006; Bell et al., 2011).

## From RNA- and protein-tagging strategies to hybrid approaches

Protein-centric and RNA-centric approaches are not redundant methods but complementary to each other, and can be great allies to map the RNA-protein interaction network. Comparison of PAR-CLIP and SILAC-based RNA pull-downs by Scheibe et al., underscores this concept very well: a strong overlap between two completely different approaches would validate them both (Scheibe et al., 2012). An iterative use of these two powerful technologies will realistically allow the systematic characterization of RNAs and RBP networks.

Additional approaches exist, but they are in general more artificial and require sophisticated target engineering systems. For instance, in the attempt to study proteins bound to IRES-containing mRNAs, Tsai et al., combined *in vivo* UV crosslink from the standard CLIP approach, with the MS2 *in vivo* Biotin Tagged RNA Affinity Purification (**MS2-BioTRAP**) to tag the RNA of interest, followed by stable isotope labeling with amino acid in cell culture (SILAC)-based quantitative mass spectrometry. The RNA of interest is tagged with the MS2 stem-loop cluster that does not hamper the normal processing and translation of the RNA (Doucet et al., 2010). The stem-loop cluster is efficiently recognized by the bacteriophage protein MS2-HB, where the HB tag consists of two hexahistidine tags, a TEV cleavage site, and a signal sequence for *in vivo* biotinylation. Therefore, capture of these complexes can be achieved simply with streptavidin beads (Tsai et al., 2011).

All protein-centric approaches obviously rely on the availability of a specific antibody to perform the immunoprecipitation step. To expand the RBP-RNA interaction landscape to those proteins for which there is no good antibody available, an alternative is the **TAG-eCLIP** (Van Nostrand et al., 2016a) where the RBP is fused to an epitope tag and then expressed as a transgene for affinity purification. Insertion of these tags into endogenous gene loci is now possible and efficient thanks to CRISPR technologies, enabling profiling of RBPs within their normal regulatory context (Ran et al., 2013). The “tag-approach” can be used with T7 epitope tag, HA or FLAG tags (i.e., FLAGK11 Matsumoto et al., 2017), and the tandem affinity purification epitope tag (TAP-tag system) (Xiong et al., 2015), that can be modified in many ways, for example substituting with a strain of histidines the sequence encoding the calmodulin binding peptide of the conventional TAP (Granneman et al., 2009), providing multiple purification options.

FLAG-HA tags or TAP tags can be inserted in expression vectors and the overexpressed tagged protein would be efficiently purified and enriched, overcoming one of the main practical constrains of mass spectrometry, the high amount of material required for *in vivo* purifications (Baltz et al., 2012). A main challenge for MS studies is that proteins, unlike DNA or RNA, cannot be amplified by current technologies and this is still the Achilles' heel of mass spectrometry approach. As a matter of fact, despite the big advances in mass spectrometry technology, there are still limitations to this technique, especially due to the dynamic range limitations in the mass spectrometer in the presence of highly abundant unspecific background binders (Scheibe et al., 2012), therefore optimization and appropriate controls and replicates are fundamental for a solid analysis (McHugh et al., 2014). High-resolution nano-LC MS/MS and protein identification can be maximized with peptide fractionation using isoelectric focusing, this step will reduce sample complexity and improve detection efficiency (Castello et al., 2015). A solid but more expensive approach is the previously mentioned SILAC approach (Baltz et al., 2012). SILAC determines the intensity of specific “light” and “heavy” peptide pairs that are mass shifted from each other due to metabolic labeling of the proteome with isotope enriched amino acids (Mann, 2006). With this approach, the elicited control is generally to reverse the “light” and “heavy” samples, therefore a candidate is trustworthy if is picked either when is “light” labeled or “heavy” labeled.

A very recent hybrid approach is crosslinking of segmentally isotope-labeled RNA and tandem mass spectrometry (**CLIR-MS/MS**), to precisely identify the RNA interface of RBP and its localization on the target RNA (Dorn et al., 2017). Instead of labeling proteins, in this approach RNA is labeled with ^13^C^15^N, RNA-protein complexes get UV crosslinked and digested with trypsin and non-specific nucleases to generate peptides with short nucleotide chains adducts that can be identified by MS/MS.

Pioneer approaches to establish RNA interactomes were initially performed on abundant RNA species such as polyA-mRNAs, and were then successfully extended to tag-overexpressed ones. The first landmark study introduced a systematic, unbiased, and comprehensive approach named: RNA interactome capture technique (**IC**) (Baltz et al., 2012; Castello et al., 2015). It is an improved version of PAR-CLIP approach as it consists of a photoreactive nucleotide-enhanced UV crosslinking of living cells to introduce covalent crosslinks between proteins and RNA in direct contact, while avoiding protein–protein crosslinks. Cells are lysed and polyadenylated transcripts are captured by hybridization to oligo(dT)-beads. Washes under denaturing conditions confer stringency to the following identification of interacting proteins by LC-MS/MS (Conrad et al., 2016). These efforts generated a comprehensive atlas of mRNA (strictly: poly(A)RNA)-binding proteins of a living cell, an informative snapshot of RNA biology that can be adapted to study the mRNA interactomes of other cells and organisms, or to specifically focus on certain subcellular domains. However, focusing on polyadenylated species, this approach excludes many lncRNAs. Moreover, the main drawback encountered performing IC on poly(A)RNAs, was the persistence of DNA contaminations that can generate erroneous readouts. To improve the quality of the RNA purified, in the serial RNA Interactome Capture (**serIC**) the authors combined a rigorous cell fractionation, with serial purification steps of the RNA of interest (Conrad and Ørom, 2017), successfully increasing the RNA-RBP recovery rate.

Finally more recent studies used Halo-tags (Brannan et al., 2016) or aptamers (Butter et al., 2009) on the RNA molecule of interest to efficiently perform pull down and mass spectrometry, extending such techniques not only to any RNA of interest but importantly making it suitable for large scale screenings.

## *In silico* approaches

Prediction methods and computational models based on sequence specific RNA-protein recognition can be exploited to understand the basics of molecular recognition, to expand the RBP repertoire and discover also non-canonical RBPs. RNA-binding domains tend to show sequence and/or structural specificities, therefore different computational approaches can be used. The regulatory sequence analysis tools (RSAT) for example, is specifically designed for the detection of regulatory signals in non-coding sequences, and considers mainly the *sequences* of binding sites. Other tools can *model* binding sites in a supervised or unsupervised way, considering or ignoring the RNA structure and/or the RNA context (Re et al., 2014).

An improved approach is **catRAPID** (“fast predictions of RNA and protein interactions and domains at the Center for Genomic Regulation, Barcelona, Catalonia”), an algorithm developed to evaluate the interaction propensities of polypeptides and nucleotide chains, using their physicochemical properties instead of sequence similarity searches (Livi et al., 2015). Complex proteins and/or long RNA molecules generally give rise to less accurate predictions; catRAPID presents a *suite* to specifically handle them. It is therefore a very versatile tool that can be applied to both coding and non-coding RNAs (Bellucci et al., 2011; Marchese et al., 2016).

**Global Score** is a new algorithm recently released, to work specifically with large transcripts. Calibrated on preexisting high throughput CLIP data, Global Score can predict global and local interactions between RBP and lncRNA, and most importantly it can prioritize lncRNAs partners facilitating the design of new experiments and their validation (Cirillo et al., 2016).

Finally, one last powerful computational approach is a classification algorithm Support Vector Machine obtained from neighborhood associated RBPs (**SONAR**). It was developed modeling experimental data obtained from a small scale proteomic study where 12 Halo-Tagged RBPs were pulled down and their protein interactors analyzed with Multidimentional Protein Identification Technology (MudPIT) mass spectrometry. By comparing an RNAse treated vs. an untreated sample they could distinguish RNA-dependent interactions, therefore SONAR can discover uncharacterized candidate RBPs by leveraging protein-protein interactomes (Brannan et al., 2016).

## Conclusions and future directions

Mounting evidence from *in vitro* and *in vivo* studies supports a link between lncRNA deregulation and disease development and progression. Therefore, a deeper knowledge of the genome dark side promises to better understand the basis of disease and help the design of novel efficient therapies. Undoubtedly, the recently development of high throughput techniques for the study of RNA-protein interactions will be instrumental to unravel lncRNA mechanism of action. RNA molecules however, have the unique ability to bind other nucleic acids and/or form secondary structures within the same transcript by base-pairing. RNA secondary and tertiary structures are essential for RNA stability but also for its function, since they may create docking sites for specific RBPs. Although we have started to appreciate and explore this additional layer of complexity only recently, the study of RNA structure could unravel sets of proteins that interact with a certain ncRNA mainly via structural interface. In the future combination of interactome studies with structural models will be a powerful tool to dissect the molecular functions of lncRNAs. Another challenge for the years to come will be the genome wide-analysis of RNA-protein interaction at a single cell level. Representing an average of all the cell in a given sample, bulk RNA sequencing often masks the diversity within different subpopulations. Given the abovementioned lineage/tissue/stimulus-specific expression of lncRNAs, it would not be surprising to find out that in some cases the low levels simply reflect the expression of the lncRNA in rare cell populations (e.g., cancer stem cells). Moreover, multiple lncRNA-protein complexes might co-exist in different cells. Hence, being able to study lncRNA-protein interactions in single cells with spatial resolution, would undoubtedly improve our knowledge of lncRNA biology. A combination of immunocytochemistry and spatial transcriptomic (Ståhl et al., 2016) would be instrumental in this sense, paving the way to the study of RNA-protein interaction heterogeneity in physiological and disease condition.

## Author contributions

JB wrote the text. EL proposed the topic, selected the literature and reviewed the text.

### Conflict of interest statement

The authors declare that the research was conducted in the absence of any commercial or financial relationships that could be construed as a potential conflict of interest.
